# Dietary Vitamin D Insufficiency Aggravates High-Fat Diet-Induced Hepatic Steatosis Associated with Reduced Hepatic SIRT1 Activity and Nrf2-Related Antioxidant Gene Expression in Male C57BL/6J Mice

**DOI:** 10.3390/nu18152481

**Published:** 2026-07-31

**Authors:** Amanzholova Kamila, Eugene Chang

**Affiliations:** Department of Food and Nutrition, Kangwon National University at Gangneung, Gangneung-si 25457, Gangwon-do, Republic of Korea

**Keywords:** vitamin D, obesity, nonalcoholic fatty liver disease, sirtuin 1, nuclear factor erythroid 2–related factor 2

## Abstract

**Background/Objectives**: As obesity continues to increase globally, it has become a major contributor to the growing burden of metabolic diseases, including type 2 diabetes mellitus, cardiovascular disease, and nonalcoholic fatty liver disease (NAFLD). Hepatic lipid accumulation during obesity contributes to oxidative stress and alterations in mitochondrial homeostasis, key features of NAFLD progression. This study investigated whether vitamin D insufficiency aggravates obesity-related hepatic steatosis in association with changes in hepatic sirtuin 1 (SIRT1) activity and nuclear factor erythroid 2–related factor 2 (Nrf2)-related antioxidant gene expression. **Methods**: Male C57BL/6J mice were maintained on one of three dietary regimens for 16 weeks: a normal diet (NOR; 10% fat containing 1000 IU vitamin D/kg), a high-fat diet (HF; 60% fat containing 1000 IU vitamin D/kg), or a vitamin D-deficient high-fat diet (HF + NVD; 60% fat with no added vitamin D). **Results**: Vitamin D insufficiency in HF-fed mice (HF + NVD) was associated with significantly higher hepatic triglyceride accumulation and lipid peroxidation than those observed in the HF group. A concomitant reduction in the expression of Nrf2-dependent antioxidant genes was observed in the HF + NVD group. Furthermore, hepatic mitochondrial DNA content as well as hepatic SIRT1 mRNA level and activity, the NAD^+^/NADH ratio, and CPT1α mRNA expression were significantly decreased in the HF + NVD group. **Conclusions**: An inadequate vitamin D status was associated with greater hepatic lipid deposition, enhanced oxidative stress, and reduced hepatic SIRT1 activity, and lower expression of Nrf2-related antioxidant genes. These findings support that maintaining adequate vitamin D status might help preserve hepatic metabolic homeostasis during obesity.

## 1. Introduction

Obesity is a chronic and multifactorial metabolic disorder characterized by excessive energy storage resulting from a sustained imbalance between energy intake and expenditure [1]. Once adipose tissue reaches its storage limit, excess lipids are diverted to peripheral tissues, where lipid accumulation in organs such as the liver, skeletal muscle, and pancreas contributes to metabolic impairment. [2,3]. Nonalcoholic fatty liver disease (NAFLD) is recognized as the predominant chronic liver disorder in individuals with obesity and commonly coexists with insulin resistance, type 2 diabetes mellitus, and cardiovascular disease. [4,5]. Moreover, increasing adiposity is accompanied by a greater risk of NAFLD progression and more severe hepatic manifestations, underscoring obesity-induced lipid accumulation in the liver as a key contributor to metabolic disease [6,7,8]. Recently, NAFLD has been redefined as metabolic dysfunction-associated steatotic liver disease (MASLD) according to an international multisociety consensus, reflecting the central role of metabolic dysfunction in the disease process [9]. Therefore, elucidating the mechanisms underlying obesity-induced hepatic lipid accumulation may facilitate the identification of novel preventive and therapeutic approaches for obesity-associated metabolic disorders.

Excessive hepatic lipid accumulation promotes mitochondrial dysfunction and oxidative stress, thereby activating endogenous antioxidant defense mechanisms that are primarily coordinated by nuclear factor erythroid 2-related factor 2 (Nrf2), a key transcription factor regulating antioxidant and cytoprotective gene expression [10,11]. Once activated, Nrf2 strengthens endogenous antioxidant defenses by coordinating a broad cytoprotective gene program, thereby attenuating oxidative injury and preserving hepatic lipid and redox homeostasis [11,12,13]. This coordinated antioxidant response is supported by experimental studies showing that pharmacological or genetic activation of the Nrf2 signaling pathway decreases hepatic lipid accumulation and oxidative stress [14,15], whereas Nrf2 deficiency accelerates hepatic inflammation, fibrosis, and liver injury in dietary models of NAFLD [16], highlighting the pivotal role of Nrf2 in maintaining hepatic metabolic homeostasis. Given its pivotal role in maintaining hepatic redox homeostasis, Nrf2 activity is regulated, at least in part, by sirtuin 1 (SIRT1), an NAD^+^-dependent deacetylase that coordinates antioxidant defense with mitochondrial and metabolic homeostasis. By enhancing Nrf2 transcriptional activity, SIRT1 promotes the expression of antioxidant genes and reinforces cellular protection against oxidative stress [17,18]. Accumulating evidence suggests that coordinated SIRT1–Nrf2 signaling plays an important role in preserving mitochondrial integrity, maintaining redox balance, and regulating hepatic metabolic homeostasis, making this pathway an attractive candidate for therapeutic intervention in obesity-associated NAFLD.

In light of the proposed role of the SIRT1–Nrf2 axis in preserving hepatic mitochondrial function and redox homeostasis, interest in nutritional factors capable of regulating this pathway has increased, and vitamin D has emerged as a candidate regulator of hepatic metabolism. Supporting this concept, epidemiological studies have consistently demonstrated lower circulating 25-hydroxyvitamin D (25(OH)D) concentrations in individuals with obesity, with reduced vitamin D status associated with an increased risk of type 2 diabetes mellitus, NAFLD, and cardiovascular disease [19,20]. Clinical evidence further indicates that inadequate vitamin D status is independently associated with both occurrence and severity of NAFLD. In addition, intervention studies have reported beneficial effects of vitamin D supplementation, including reduced hepatic lipid accumulation, improved markers of liver function, and amelioration of metabolic disturbances [21,22,23]. Despite accumulating clinical evidence supporting the beneficial effects of vitamin D in obesity-associated liver disease, the molecular mechanisms underlying its hepatoprotective actions remain incompletely understood. Evidence from experimental models indicates that dietary vitamin D supplementation alleviates hepatic damage associated with high-fat (HF) feeding while improving lipid metabolism, insulin sensitivity, and oxidative stress [24,25,26]. Mechanistic studies further suggest that these beneficial effects are associated, at least in part, with coordinated regulation of the SIRT1–Nrf2 signaling axis. In palmitate-treated hepatocytes, vitamin D enhances SIRT1 activity, whereas its anti-fibrotic actions have been linked to activation of Nrf2-mediated antioxidant signaling [27]. However, most previous experimental studies have focused on the beneficial effects of vitamin D supplementation rather than the metabolic consequences of chronic dietary vitamin D insufficiency itself. Therefore, whether chronic dietary vitamin D insufficiency aggravates HF diet-induced hepatic steatosis and is accompanied by alterations in hepatic SIRT1 activity and Nrf2-related antioxidant responses in vivo remains unclear. Accordingly, this study investigated whether dietary vitamin D insufficiency is associated with aggravated HF-induced hepatic steatosis, alterations in hepatic SIRT1 activity, and Nrf2-related antioxidant gene expression in HF-fed obese mice.

## 2. Materials and Methods

### 2.1. Animals and Diets

All procedures involving animals were carried out in accordance with the international guidelines by the National Institutes of Health (NIH) and were approved by the Institutional Animal Care and Use Committee (IACUC) of Ewha Womans University (IACUC No. 19-005). A total of 27 five-week-old male C57BL/6J mice were obtained from SaeronBio, Inc. (Uiwang-si, Gyeonggi-do, Republic of Korea). Animals were maintained under ultraviolet B (UV-B) free conditions with a 12 h light/dark cycle at 22 ± 1 °C and 55 ± 5% relative humidity, with two to three mice housed in each cage. Nesting material was supplied to provide environmental enrichment. Following a 7-day acclimation period with unrestricted access to water and a normal diet, mice were allocated by simple randomization into three experimental groups (*n* = 9 per group): (1) a normal diet group receiving 10% fat containing 1000 IU vitamin D/kg (NOR; D12450), (2) a high-fat diet group with 60% fat containing 1000 IU vitamin D/kg (HF; D12492), or (3) a high-fat diet group without vitamin D (HF + NVD). All diets were obtained from Research Diets, Inc. (New Brunswick, NJ, USA). Based on the National Research Council (NRC) recommendation of 1000 IU vitamin D/kg diet for laboratory rodents, mice in the NOR and HF groups received the recommended vitamin D level, whereas the HF + NVD group received a vitamin D-free diet (0 IU/kg). This dietary design was intended to establish distinct vitamin D status while minimizing nonspecific adverse effects unrelated to vitamin D insufficiency. Body weight and food intake were monitored and measured throughout the 16-week feeding period at weekly intervals. Upon completion of the intervention, mice were fasted overnight before euthanasia by CO_2_ inhalation. Whole blood was collected by cardiac puncture, and serum was separated by centrifugation (2000× *g* for 20 min, 4 °C) before storage at −70 °C. Liver tissues were immediately excised, snap-frozen in liquid nitrogen, and preserved at −70 °C until subsequent analyses.

### 2.2. Serum 25-Hydroxy Vitamin D (25(OH)D) Analysis

Serum 25(OH)D concentrations were determined using a commercial enzyme-linked immunosorbent assay (ELISA) kit (Eagle Biosciences, Inc., Amherst, NH, USA) according to the supplier’s instructions. The assay is based on a competitive immunoassay format in which endogenous 25(OH)D and a biotin-conjugated vitamin D tracer compete for antibody binding sites. Following the binding reaction and washing procedures, enzyme substrate was added to produce a color signal, and optical density was measured at 450 nm using a Varioskan Flash microplate reader (Thermo Scientific, Waltham, MA, USA). Because this assay follows a competitive format, absorbance is inversely associated with the serum concentration of 25(OH)D.

### 2.3. Measurement of Serum Metabolic Parameters

Serum alanine aminotransferase (ALT) and aspartate aminotransferase (AST) activities were analyzed by enzyme-based colorimetric assays using reagent kits from Sigma-Aldrich (St. Louis, MO, USA). Serum triglyceride (TG) and total cholesterol (TC) concentrations were quantified with commercial assay kits (Abcam, Cambridge, UK). Serum high-density lipoprotein cholesterol (HDL-C) concentrations were measured using diagnostic reagents supplied by Asan Pharmaceutical Co., Ltd. (Seoul, Republic of Korea), whereas low-density lipoprotein cholesterol (LDL-C) concentrations were estimated according to the Friedewald equation (LDL-C = TC − HDL-C − (TG/5)) [28].

### 2.4. Hepatic Lipid Content Analysis

Total lipids were extracted from liver samples using a modified protocol based on the method of Bligh and Dyer [29]. Approximately 0.5 g of liver tissue was suspended in 1.5 mL of 0.9% saline, subsequently mixed with 7.5 mL of a chloroform: methanol mixture (1:2, *v*/*v*). The homogenate was maintained for about 1 h, followed by supplementation with 2.5 mL of chloroform to achieve organic phase separation. After centrifugation (3000 rpm, 20 min), the lower solvent layer containing lipids was transferred to a clean tube. Following solvent evaporation, the extracted lipid residue was reconstituted in an *n*-hexane: isopropanol solution (3:2, *v*/*v*) and stored at −30 °C until further analysis. Hepatic concentrations of TC and TG were then determined using enzymatic assay kits as previously described.

### 2.5. Determination of Serum Lactate Dehydrogenase (LDH) Levels

Serum lactate dehydrogenase (LDH) levels were determined using a commercially available colorimetric assay kit (Abcam, Cambridge, UK) in accordance with the manufacturer’s protocol. The assay utilizes a NAD-dependent enzyme-coupled reaction to generate a chromogenic product proportional to LDL contents in the sample. After completion of the enzymatic reaction, absorbance was recorded at 450 nm using a Varioskan Flash microplate reader (Thermo Scientific, Waltham, MA, USA). Serum LDH values were expressed as fold change relative to the HF group, with the HF group serving as the reference (fold = 1.0).

### 2.6. Measurement of Hepatic Lipid Peroxidation

To evaluate hepatic lipid peroxidation, malondialdehyde (MDA) levels were quantified using a commercially available thiobarbituric acid reactive substances (TBARS) colorimetric assay kit (Abcam, Cambridge, UK) in accordance with the manufacturer’s instructions. Liver was homogenized in lysis buffer supplemented with butylated hydroxytoluene to minimize artificial lipid oxidation. Following centrifugation (13,000× *g*, 10 min, 4 °C), the clarified supernatant was subjected to thiobarbituric acid (TBA), and the resulting MDA-TBA adduct was quantified by measuring absorbance at 532 nm using a microplate reader (Thermo Scientific, Waltham, MA, USA). After normalization to protein content quantified by a bicinchoninic acid (BCA) assay (Thermo Scientific, Waltham, MA, USA), hepatic MDA levels were expressed as fold change relative to the HF group, with the HF group serving as the reference (fold = 1.0).

### 2.7. Histological Analysis

Liver specimens were prepared for histological examination by fixation in 10% formalin for 24 h, followed by paraffin embedding and sectioning at a thickness of 5 μm. The tissue sections were stained with hematoxylin and eosin (H&E) using standard histological procedures. Histopathological alterations, including changes in hepatocellular morphology, lipid accumulation, and tissue architecture, were qualitatively evaluated using an Olympus light microscope (Tokyo, Japan) at 400× magnification.

### 2.8. Transmission Electron Microscopy (TEM)

For ultrastructural evaluation, representative liver specimens were selected from each experimental group and immediately immersed in a primary fixative containing 2% glutaraldehyde and paraformaldehyde prepared in 0.1 M phosphate buffer (pH 7.4). Primary fixation was carried out for 2 h at room temperature to preserve cellular and subcellular architecture. The specimens were subsequently subjected to 1% osmium tetroxide in the same buffer for 1 h. After post-fixation, the samples were progressively dehydrated using increasing concentrations of ethanol and embedded in epoxy resin. Ultrathin sections were obtained from the resin-embedded tissue blocks, contrasted with appropriate electron-dense stains, and visualized using a transmission electron microscope (TEM; Hitachi, Tokyo, Japan). Mitochondrial morphology and ultrastructural integrity were qualitatively evaluated from the acquired electron micrographs.

### 2.9. Hepatic Mitochondrial DNA (mtDNA) Measurement

Liver tissue DNA was extracted using the Gentra Puregene DNA Isolation Kit (Qiagen, Valencia, CA, USA) in accordance with the manufacturer’s protocol. The relative mitochondrial DNA (mtDNA) content was assessed by real-time quantitative PCR (RT-qPCR) through simultaneous amplification of a mitochondrial target gene and a nuclear reference gene. Cytochrome c oxidase subunit 1 (COX1) was selected as the mitochondrial marker, whereas glyceraldehyde 3-phosphate dehydrogenase (GAPDH) served as the nuclear reference. Relative mtDNA copy number was calculated from the COX1/GAPDH ratio and used as an indicator of mitochondrial DNA abundance in liver tissue.

### 2.10. Real-Time Quantitative Polymerase Chain Reaction (RT-qPCR)

Total RNA was purified from liver samples using the RNeasy Plus Mini Kit (Qiagen, Hilden, Germany). The quantity and purity of the recovered RNA were evaluated spectrophotometrically with a NanoDrop1000 spectrophotometer (Thermo Scientific, Waltham, MA, USA). For each sample, 1 μg of RNA was converted into complementary DNA using the Moloney Murine Leukemia Virus (MMLV) Reverse Transcriptase Kit (Bioneer, Daejeon, Republic of Korea). Reverse transcription was conducted at 37 °C for 60 min, followed by enzyme inactivation at 95 °C for 5 min. Quantitative PCR amplification was subsequently performed with AccuPower 2X GreenStar qPCR Master Mix (Bioneer, Daejeon, Republic of Korea) using a Rotor-Gene Q real-time PCR platform (Qiagen, Valencia, CA, USA). The thermal program consisted of an initial denaturation step at 95 °C for 10 min, followed by 40 amplification cycles comprising denaturation at 95 °C for 15 s, primer annealing at 60 °C for 20 s, and extension at 72 °C for 20 s. Relative mRNA expression was normalized to β-actin and quantified using the comparative Ct (2^−∆∆CT^) [30]. Hepatic mRNA expression was expressed as fold change using the HF group as the reference. Supplementary Table S1 provides the sequences of the primers used in the RT-qPCR analysis.

### 2.11. Hepatic SIRT1 Activity Measurement

Hepatic SIRT1 activity was evaluated in nuclear extracts with a fluorometric assay system from Abcam (Cambridge, UK). Samples were incubated with an acetylated fluorescent substrate, NAD^+^, and trichostatin A. Following SIRT1-mediated deacetylation, the developer solution containing lysylendopeptidase was introduced to generate fluorescence from the deacetylated substrate. After termination of the reaction with the supplied 2X stop reagent, fluorescence was detected at 340 nm excitation and 460 nm emission on a Thermo Scientific microplate platform (Waltham, MA, USA). Total protein in each sample was measured by the BCA protein assay kit (Thermo Scientific, Waltham, MA, USA) and SIRT1 activity was adjusted accordingly. Final values were reported as fold differences relative to the HF group.

### 2.12. Measurement of Hepatic NAD^+^/NADH Ratio

Hepatic NAD^+^ and NADH levels were quantified using a commercially available NAD^+^/NADH assay kit (Abcam, Cambridge, UK). Frozen liver specimens were homogenized in the extraction solution supplied with the assay kit to minimize nucleotide degradation. The resulting lysates were then processed according to the manufacturer’s assay procedure. Total protein in each sample was independently quantified by the BCA method (Pierce, Rockford, IL, USA), and NAD^+^ and NADH values were normalized to the corresponding protein content. The NAD^+^/NADH ratio was subsequently calculated, and all values were expressed as fold changes compared with the HF group.

### 2.13. Statistical Analysis

All experimental measurements are reported as mean ± standard error of the mean (SEM). Data analysis was carried out using IBM SPSS Statistics version 32.0 (IBM Corp., Armonk, NY, USA). Statistical analyses were performed using predefined pairwise comparisons according to the study design. Differences between two groups were examined using two-tailed Student’s *t*-tests, with statistical significance established at *p* < 0.05.

## 3. Results

### 3.1. Impact of Vitamin D-Deficient Diet on Body Weight and Fat Deposition in HF-Fed Obese Mice

**Figure 1:**
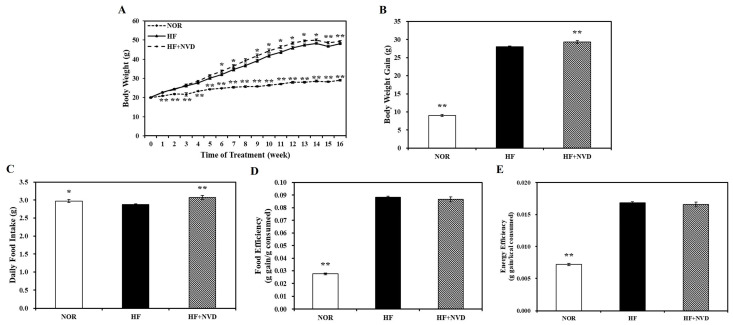
Effects of dietary vitamin D insufficiency on obesity in mice fed experimental diets for 16 weeks. (**A**) Time course of body weight (g), (**B**) body weight gain (g), (**C**) daily food intake, (**D**) food efficiency (g weight gain/g food consumed), and (**E**) energy efficiency (g weight gain/kcal consumed). Values are expressed as mean ± SEM (*n* = 9/group). NOR, 10% fat diet containing 1000 IU vitamin D; HF, 60% fat diet containing 1000 IU vitamin D; HF + NVD, 60% fat diet without vitamin D supplementation. * *p* < 0.05; ** *p* < 0.01 versus the HF group.

After a one week of adaptation period, male C57BL/6J mice (6 weeks of age) were randomly assigned to one of three dietary regimens for 16 weeks (*n* = 9 per group): a 10% fat diet containing 1000 IU vitamin D/kg (NOR), a 60% fat diet containing 1000 IU vitamin D/kg (HF), or a high-fat diet lacking vitamin D (HF + NVD) for 16 weeks. Baseline body weight was comparable among the three groups before dietary intervention, with no statistically significant differences observed (Figure 1A). A significant increase in BW was observed in HF-fed mice compared to NOR-fed controls beginning at week 1 (*p* < 0.05), with final BW and total BW gain significantly higher in the HF group (*p* < 0.01; Figure 1A,B), confirming the development of diet-induced obesity. Final body weight and cumulative body weight gain were significantly higher in the HF + NVD group than in the HF group (Figure 1A,B). Differences in body weight trajectories emerged after week 6, supporting the notion that insufficient vitamin D levels in the HF diet enhanced susceptibility to diet-induced weight gain. Although the HF + NVD group exhibited a higher mean daily food intake than the HF group, food efficiency and energy efficiency did not differ significantly between the two groups. (Figure 1C–E).

### 3.2. Changes in Serum 25-Hydroxyvitamin D Levels Following Dietary Vitamin D Insufficiency

**Figure 2:**
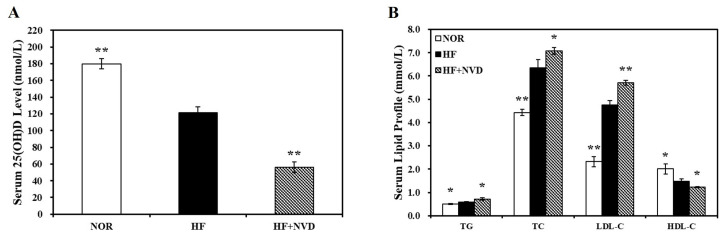
Effects of dietary vitamin D insufficiency on circulating 25-hydroxy vitamin D and serum lipid parameters. (**A**) Serum 25-hydroxyvitamin D (25(OH)D) concentrations. (**B**) Serum triglyceride (TG), total cholesterol (TC), high-density lipoprotein cholesterol (HDL-C), and low-density lipoprotein cholesterol (LDL-C). LDL-C was calculated as TC − HDL-C − (TG/5). Values are expressed as mean ± SEM (*n* = 9/group). NOR, 10% fat diet containing 1000 IU vitamin D; HF, 60% fat diet containing 1000 IU vitamin D; HF + NVD, 60% fat diet without vitamin D supplementation. * *p* < 0.05; ** *p* < 0.01 versus the HF group.

Dietary vitamin D insufficiency markedly altered systemic vitamin D status, as reflected by circulating serum 25(OH)D concentrations. Serum 25(OH)D levels declined markedly in response to HF feeding and were lowest in mice receiving the vitamin D-deficient HF diet (Figure 2A). Compared with the NOR group, both HF and HF + NVD mice exhibited significantly reduced serum 25(OH)D concentrations, with the greatest reduction observed in the HF + NVD group versus the HF group (*p* < 0.01). According to the Endocrine Society, vitamin D deficiency is defined as a serum 25(OH)D concentration < 20 ng/mL (<50 nmol/L), whereas vitamin D insufficiency is defined as 20–29 ng/mL (50–74 nmol/L) [31,32]. Despite a significant reduction, the mean serum 25(OH)D concentration in the HF group remained within the vitamin D-sufficient range (121.54 ± 6.58 nmol/L), whereas the HF + NVD group exhibited a mean serum 25(OH)D concentration of 56.19 ± 6.42 nmol/L. These results indicate successful establishment of a diet-induced vitamin D insufficiency model.

Serum lipid parameters were assessed to determine whether dietary vitamin D insufficiency influences obesity-related dyslipidemia (Figure 2B). HF feeding markedly altered the serum lipid profile, resulting in 13.9%, 43.1%, and 104.5% increases in circulating TG, TC, and LDL-C concentrations, respectively (*p* < 0.01) and a 26.3% decrease in serum HDL-C concentrations compared with the NOR group (*p* < 0.05). The dyslipidemic alterations induced by HF feeding became more pronounced following dietary vitamin D insufficiency. Compared with the HF group, serum TG, TC, and LDL-C concentrations increased by a further 23.4%, 11.5%, and 20.2%, respectively, whereas HDL-C concentrations decreased by an additional 16.9% (*p* < 0.05).

### 3.3. Vitamin D Insufficiency Aggravates High-Fat Diet-Induced Hepatic Fat Deposition and Lipid Peroxidation

**Figure 3:**
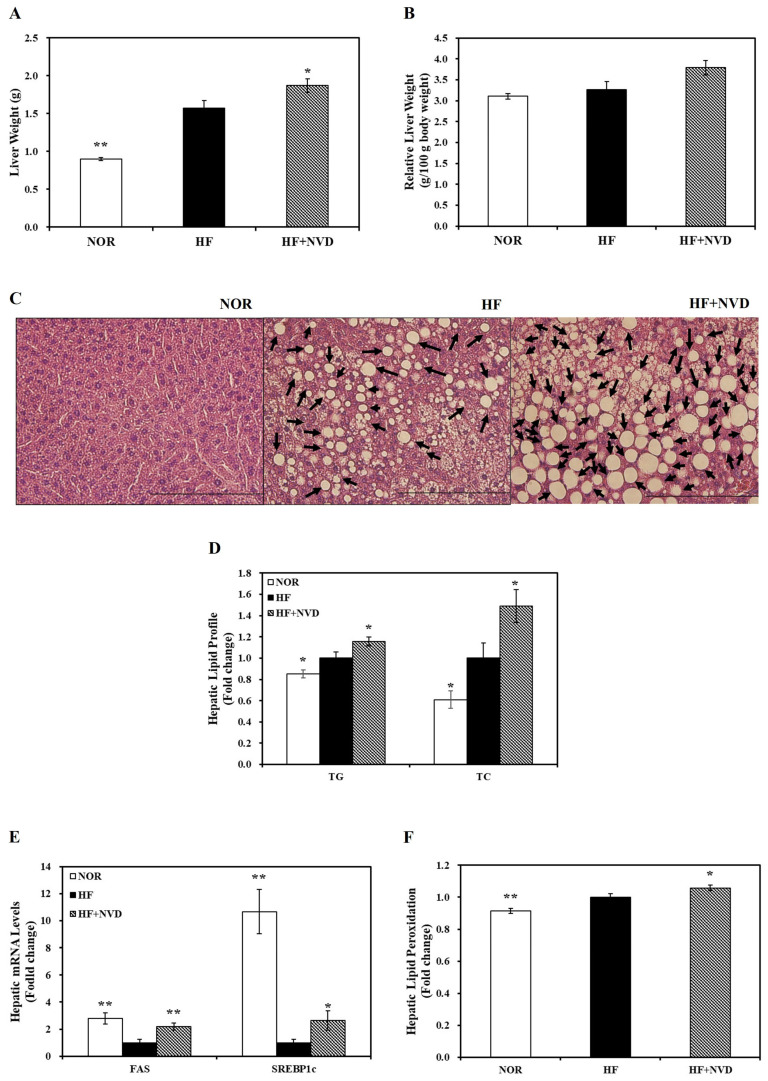
Dietary vitamin D insufficiency aggravates hepatic lipid accumulation and lipid peroxidation in high-fat diet-fed mice. (**A**) Liver weight. (**B**) Relative liver weight (g/100 g body weight). (**C**) Representative H&E-stained liver sections (400×; scale bars = 200 µm). Black arrows indicate lipid droplets. (**D**) Hepatic triglyceride (TG) and total cholesterol (TC) contents. (**E**) Relative hepatic mRNA expression of FAS and SREBP-1c. (**F**) Hepatic malondialdehyde (MDA) concentrations. Data are presented as mean ± SEM (*n* = 9/group). NOR, 10% fat diet containing 1000 IU vitamin D; HF, 60% fat diet containing 1000 IU vitamin D; HF + NVD, 60% fat diet without vitamin D supplementation. FAS, fatty acid synthase; SREBP-1c, sterol regulatory element-binding protein-1c. * *p* < 0.05; ** *p* < 0.01 versus the HF group.

Absolute liver weight increased progressively across the experimental groups and was significantly higher in the HF group than in the NOR group, with a further significant 19.1% increase in the HF + NVD group compared with the HF group (Figure 3A; *p* < 0.05). Relative liver weight (liver weight per 100 g body weight) also increased by 16.2% in the HF + NVD group compared with the HF group; however, this increase did not reach statistical significance (Figure 3B).

Representative H&E-stained liver sections showed preserved hepatic architecture without apparent histopathological abnormalities in the NOR group, whereas the HF group exhibited moderate lipid accumulation. In contrast, the HF + NVD group exhibited larger lipid droplets and more extensive hepatic fat deposition than the HF group (Figure 3C). Consistent with the histological observations, quantitative analysis of hepatic lipid contents demonstrated that hepatic TG and TC concentrations were 17.6% and 63.9% higher, respectively, in the HF group than in the NOR group. Compared with the HF group, dietary vitamin D insufficiency further aggravated hepatic lipid accumulation, with hepatic TG and TC concentrations increasing by 15.6% and 48.9%, respectively (Figure 3D; *p* < 0.05).

To determine whether hepatic lipid accumulation was accompanied by changes in lipogenic gene expression, hepatic mRNA levels of sterol regulatory element-binding protein-1c (SREBP-1c) and fatty acid synthase (FAS) were measured. Compared with the HF group, the HF + NVD group exhibited 2.19- and 2.64-fold higher expression levels of FAS and SREBP-1c, respectively (Figure 3E; *p* < 0.05). In parallel, hepatic MDA levels were significantly higher in the HF + NVD group than in the HF group, reflecting increased lipid peroxidation (Figure 3F; *p* < 0.05).

### 3.4. Dietary Vitamin D Insufficiency Exacerbates High-Fat Diet-Induced Liver Injury

**Figure 4:**
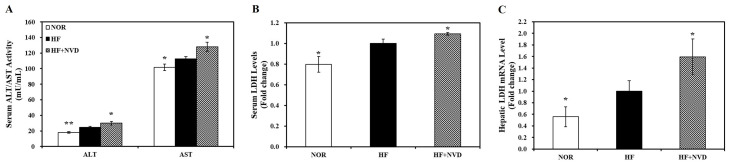
Impact of dietary vitamin D insufficiency on high-fat diet-induced hepatocellular injury. (**A**) Serum ALT and AST activities. (**B**) Serum LDH levels presented relative to the HF group. (**C**) Relative hepatic LDH mRNA expression normalized to β-actin. Data are presented as fold changes relative to the HF group and as mean ± SEM (*n* = 9/group). NOR, 10% fat diet containing 1000 IU vitamin D; HF, 60% fat diet containing 1000 IU vitamin D; HF + NVD, 60% fat diet without vitamin D supplementation. ALT, alanine transaminase; AST, aspartate aminotransferase; LDH, lactate dehydrogenase. * *p* < 0.05; ** *p* < 0.01 versus the HF group.

Next, serum ALT and AST activities were measured to determine whether vitamin D insufficiency exacerbates HF-induced hepatocellular injury after 16 weeks of dietary intervention. Compared with the NOR group, circulating ALT and AST activities were significantly increased by 35.8% and 10.4%, respectively, in the HF group. Dietary vitamin D insufficiency (HF + NVD) further elevated serum ALT and AST activities by 22.8% and 13.8%, respectively, compared with the HF group (Figure 4A; *p* < 0.05). Consistent with the increases in serum ALT and AST activities, serum LDH levels, a marker of hepatocellular damage, were significantly elevated in the HF + NVD group compared with the HF group (Figure 4B; *p* < 0.05). To further examine hepatocellular injury at the molecular level, hepatic LDH mRNA expression was analyzed. Hepatic LDH mRNA expression was further increased from 1.00-fold in the HF group to 1.59-fold in the HF + NVD group (Figure 4C; *p* < 0.05). These findings suggest that dietary vitamin D insufficiency is associated with aggravated hepatocellular injury in HF-fed mice.

### 3.5. Influence of Vitamin D Depletion in High-Fat Diet on Obesity-Related Hepatic Mitochondrial Changes

**Figure 5:**
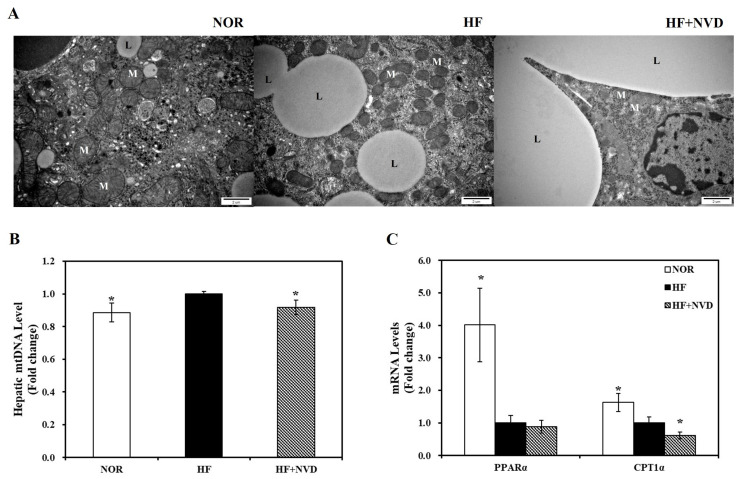
Alterations in hepatic mitochondrial morphology, mitochondrial DNA (mtDNA) content, and mitochondrial fatty acid oxidation-related gene expression. (**A**) Representative transmission electron microscopy (TEM) images of the liver tissue (20,000×; scale bars = 2 µm). L and M indicate lipid droplets and mitochondria, respectively. (**B**) Relative hepatic mtDNA content quantified by qRT-PCR. (**C**) Relative mRNA expression of mitochondrial fatty acid oxidation-related genes CPT1α and PPARα, determined by qRT-PCR, normalized to β-actin. Values are expressed as fold changes relative to the HF group and presented as mean ± SEM (*n* = 9/group). NOR, 10% fat diet containing 1000 IU vitamin D; HF, 60% fat diet containing 1000 IU vitamin D; HF + NVD, 60% fat diet without vitamin D supplementation: CPT1α, carnitine palmitoyltransferase 1α; PPARα, peroxisome proliferator-activated receptor α.* *p* < 0.05 versus the HF group.

Representative TEM images demonstrated that hepatic mitochondria in the HF + NVD group exhibited more irregular morphology and reduced cristae density than those in the HF group (Figure 5A). Hepatic mtDNA content, a marker of mitochondrial abundance, was subsequently quantified by RT-qPCR and was significantly reduced in the HF + NVD group relative to the HF group (Figure 5B; *p* < 0.05). Next, mRNA expression of genes associated with mitochondrial fatty acid metabolism was determined by RT-qPCR. Carnitine palmitoyltransferase 1α (CPT1α), a key enzyme responsible for mitochondrial fatty acid transport, was significantly downregulated by 0.61-fold in the HF + NVD group relative to the HF group (Figure 5C; *p* < 0.05). In contrast, peroxisome proliferator-activated receptor α (PPARα) mRNA expression showed a slight, non-significant decrease (Figure 5C). Collectively, these findings suggest that dietary vitamin D insufficiency is associated with alterations in hepatic mitochondrial integrity and fatty acid metabolism in HF-fed obese mice.

### 3.6. Effect of Vitamin D Insufficiency on Hepatic SIRT1-Nrf2 Antioxidant Signaling Axis

**Figure 6:**
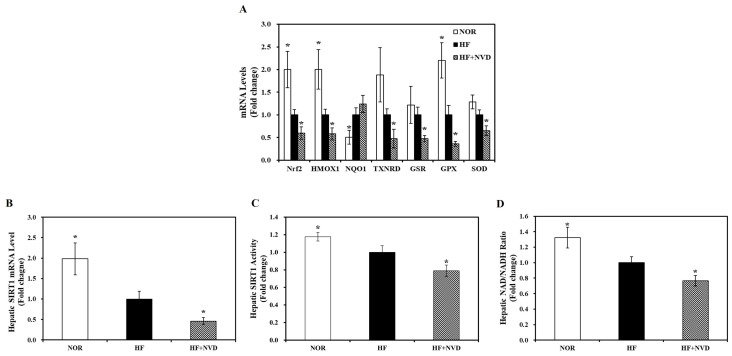
Influence of vitamin D insufficiency on hepatic antioxidant-related gene expression, SIRT1 activity, and NAD^+^/NADH ratio. (**A**) Relative mRNA expression of Nrf2 and its downstream antioxidant genes (HMOX1, NQO1, TXNRD1, GSR, GPX, and SOD) and (**B**) SIRT1 in liver tissues. (**C**) Hepatic SIRT1 activity and (**D**) NAD^+^/NADH ratio. mRNA expression levels were determined by RT-qPCR, normalized to β-actin, and expressed as fold change relative to the HF group. SIRT1 activity and the NAD^+^/NADH ratio were normalized to total protein content and expressed as fold changes relative to the HF group. Values are presented as mean ± SEM (*n* = 9/group). NOR, 10% fat diet containing 1000 IU vitamin D; HF, 60% fat diet containing 1000 IU vitamin D; HF + NVD, 60% fat diet without vitamin D supplementation. * *p* < 0.05 versus the HF group.

Dietary vitamin D insufficiency altered the mRNA expression of Nrf2 and its downstream antioxidant genes (Figure 6A). Compared with the HF group, the HF + NVD group exhibited significant reductions in hepatic mRNA expression of Nrf2 (40%), heme oxygenase 1 (HMOX1, 42%), thioredoxin reductase (TXNDR, 52%), glutathione reductase (GSR, 52%), glutathione peroxidase (GPX, 64%), and superoxide dismutase (SOD, 35%), respectively (*p* < 0.05; Figure 6A). No significant difference was observed in NAD(P)H quinone dehydrogenase 1 (NQO1) mRNA expression between the HF and HF + NVD groups. These findings suggest an association between dietary vitamin D insufficiency and reduced hepatic antioxidant gene expression during HF feeding.

To further explore the mechanisms underlying the impaired antioxidant response observed in vitamin D-insufficient obese mice, we explored hepatic SIRT1 activity and expression and NAD^+^/NADH ratio. As shown in Figure 6B, hepatic SIRT1 mRNA expression was significantly lower in the HF group than in the NOR group (*p* < 0.05). Dietary vitamin D insufficiency resulted in an additional 54% reduction in hepatic SIRT1 gene expression compared with the HF group (*p* < 0.05). Furthermore, hepatic SIRT1 enzymatic activity and the hepatic NAD^+^/NADH ratio were decreased by 18.6% and 20.0%, respectively, in the HF + NVD group relative to the HF group (*p* < 0.05; Figure 6B,C). These findings indicate lower hepatic SIRT1 expression and activity, together with a reduced NAD^+^/NADH ratio in the HF + NVD group under HF conditions.

## 4. Discussion

Hepatic steatosis represents a key manifestation of obesity-associated metabolic dysfunction and the initial stage of NAFLD progression. Although numerous epidemiological studies have linked low vitamin D status with NAFLD, direct experimental evidence regarding the metabolic consequences of dietary vitamin D insufficiency remains limited. In the present study, vitamin D insufficiency markedly aggravated hepatic lipid accumulation and lipid peroxidation in HF-induced obese mice, demonstrating that inadequate vitamin D status is associated with greater susceptibility to obesity-associated hepatic dysfunction.

Given the well-established association between vitamin D status and obesity-associated metabolic disorders, accurate assessment of vitamin D status is essential for interpreting its metabolic consequences [19,21,33]. Serum 25(OH)D concentration is widely accepted as the most reliable biomarker of vitamin D status because it reflects both dietary vitamin D intake and endogenous synthesis [34,35]. In the present study, HF feeding significantly reduced circulating 25(OH)D concentrations compared with the NOR group despite identical dietary vitamin D supplementation (1000 IU/kg diet), suggesting that obesity itself adversely affects systemic vitamin D status. Nevertheless, serum 25(OH)D concentrations in the HF group remained above 75 nmol/L, a level generally considered vitamin D sufficient. In contrast, the HF + NVD further decreased serum 25(OH)D concentrations to 22.5 ng/mL (56.2 nmol/L), which falls within the range of vitamin D insufficiency (21–29 ng/mL; 51–74 nmol/L) according to the Institute of Medicine criteria [31,32]. These findings indicate that dietary vitamin D depletion effectively shifted animals from a vitamin D-sufficient to an insufficient status based on the human clinical criteria for serum 25(OH)D concentrations. However, because HF itself significantly reduced circulating 25(OH)D concentrations, the absence of a NOR + NVD group precludes clear discrimination between the independent effects of dietary vitamin D depletion and obesity. Future studies including a NOR + NVD group are therefore warranted.

An inverse association between circulating 25(OH)D concentrations and adiposity has been consistently documented in numerous epidemiological and clinical studies [19,21,36]. A systematic review further demonstrated that central obesity, reflected by increased waist circumference, is associated with a greater risk of vitamin D deficiency and insufficiency [37,38]. Although whether low vitamin D status is a cause or a consequence of obesity remains unresolved, accumulating evidence from experimental studies suggests that vitamin D supplementation attenuates weight gain and improves metabolic abnormalities in diet-induced obesity [27,39]. Experimental studies further demonstrate that vitamin D supplementation reduces lipid accumulation and adiposity while improving systemic metabolic homeostasis across multiple obesity models [27,40,41,42,43]. Consistent with these observations, vitamin D insufficiency in the present study further increased body weight in HF-fed mice. These findings suggest that inadequate vitamin D status may be associated with impaired metabolic regulation and greater susceptibility to weight gain during 16 weeks of HF feeding. Interestingly, vitamin D-insufficient mice consumed significantly more food than HF-fed controls, whereas food efficiency and energy efficiency did not differ between the two groups. These findings suggest that the greater body weight observed in the HF + NVD group may be primarily attributable to increased food intake rather than enhanced metabolic efficiency. Future pair-feeding studies will be valuable to determine whether the greater weight gain associated with dietary vitamin D insufficiency is driven primarily by increased food intake or by vitamin D-dependent alterations in whole-body metabolism.

Hepatic steatosis is accompanied by progressive hepatic oxidative stress and dysregulated lipid metabolism during obesity [7], prompting us to investigate whether vitamin D insufficiency further aggravates these pathological alterations. Serum ALT and AST activities, established biomarkers of hepatocellular injury [44], were significantly elevated in vitamin D-insufficient HF-fed mice, indicating aggravated liver damage. This observation is supported by clinical evidence demonstrating an inverse association between circulating 25(OH)D concentrations and liver dysfunction [21]. Individuals with elevated ALT levels have been reported to exhibit significantly lower serum vitamin D concentrations, and hypovitaminosis D is highly prevalent among adolescents with suspected NAFLD defined by elevated ALT levels [45,46]. Furthermore, a large retrospective cohort study involving more than 180,000 Korean adults demonstrated that lower serum 25(OH)D concentrations were associated with a significantly greater risk of developing NAFLD, whereas higher vitamin D levels were linked to disease improvement [47].

Consistent with these clinical observations, vitamin D insufficiency was associated with more pronounced circulating lipid abnormalities, hepatic lipid accumulation and a significant increase in absolute liver weight in HF-fed obese mice. Although relative liver weight (g liver/100 g body weight) showed only a nonsignificant tendency to increase, this finding likely reflects the greater increase in body weight relative to liver weight in the HF + NVD group rather than a lack of hepatic lipid accumulation. Indeed, hepatic triglyceride content, histological fat deposition, and liver injury markers consistently indicated a more severe hepatic phenotype under vitamin D-insufficient conditions. Similar associations between vitamin D deficiency and unfavorable serum lipid profiles have been reported in clinical studies [48,49], while HF-induced obese rodents exhibit increased liver weight, hyperlipidemia, liver dysfunction, and reduced circulating 25(OH)D concentrations compared with control animals [41,50]. Elevated hepatic MDA levels further suggest enhanced lipid peroxidation under vitamin D-insufficient conditions, consistent with in vitro evidence showing that vitamin D supplementation attenuates palmitic acid-induced lipid accumulation while reducing MDA contents in C2C12 cells [42]. Taken together, these findings suggest that vitamin D insufficiency may aggravate hepatic lipid accumulation and oxidative injury during the early progression of obesity-associated NAFLD. Future studies incorporating pair-feeding strategies and detailed body composition analyses will be valuable to clarify the relative contributions of increased body weight and hepatic enlargement to liver weight changes associated with dietary vitamin D insufficiency.

To further characterize the molecular alterations associated with hepatic lipid dysregulation, we examined key regulators of hepatic lipid metabolism. Vitamin D insufficiency significantly increased the hepatic mRNA expression of the lipogenic regulators SREBP-1c and FAS while suppressing CPT1α expression, a transcriptional pattern consistent with enhanced de novo lipogenesis and reduced expression of a key fatty acid β-oxidation-related gene [51,52]. These transcriptional changes may help explain the aggravated dyslipidemia and hepatic steatosis observed in the present study. Supporting this interpretation, vitamin D supplementation has been shown to attenuate hepatic lipid accumulation through suppression of lipogenic pathways, including SREBP-1c and FAS, in type 2 diabetic mice [50]. Likewise, vitamin D insufficiency downregulates key fatty acid β-oxidation-related genes, including CPT1α, PPARα, and PGC1α, and exacerbates body weight gain and adipose tissue fat deposition in diet-induced obese rats [41]. Together, these findings suggest that inadequate vitamin D status is associated with a hepatic transcriptional profile favoring lipogenesis over fatty acid β-oxidation, which may contribute to hepatic lipid accumulation during dietary obesity.

Beyonds these alterations in hepatic lipid accumulation, mitochondrial adaptation is essential for maintaining hepatic lipid homeostasis under conditions of metabolic overload, as mitochondria serve as the primary site of fatty acid β-oxidation and oxidative phosphorylation [53]. Excessive hepatic lipid accumulation increases mitochondrial reactive oxygen species (ROS) production, leading to oxidative stress, mtDNA damage, and progressive impairment of mitochondrial function that have been associated with NAFLD progression [54]. Consistent with this concept, hepatic mtDNA content was significantly increased in HF-fed mice compared with the NOR group, which may reflect a compensatory increase in mitochondrial content or biogenesis in response to lipid overload. In contrast, hepatic mtDNA content was significantly reduced in vitamin D-insufficient HF-fed mice, suggesting attenuated mitochondrial adaptation under conditions of metabolic stress. Although NRF-1 and TFAM mRNA expression showed a similar downward trend without reaching statistical significance, the reduction in mtDNA content may reflect alterations in mitochondrial homeostasis and increased susceptibility to oxidative damage under vitamin D-insufficient conditions. However, because mitochondrial respiration and oxidative phosphorylation were not directly measured, these findings should not be interpreted as definitive evidence of impaired mitochondrial function. Future studies incorporating direct assessments of mitochondrial function, including mitochondrial respiration, oxidative phosphorylation, ATP production, mitochondrial ROS generation, together with quantitative analyses of mitochondrial ultrastructural integrity, will be necessary to determine whether the observed alterations in mtDNA content and mitochondrial biogenesis-related gene expression are accompanied by functional mitochondrial impairment under conditions of dietary vitamin D insufficiency. Taken together, these findings indicate that vitamin D insufficiency is associated with alteration in molecular and morphological markers related to mitochondrial homeostasis during chronic metabolic stress. These concurrent alterations may reflect impaired mitochondrial adaptation and are consistent with the more severe hepatic phenotype observed in obese mice with vitamin D insufficiency.

Given the central role of SIRT1 in regulating mitochondrial homeostasis and antioxidant defense during obesity-associated hepatic metabolic stress, we next investigated whether vitamin D insufficiency alters hepatic SIRT1 activity. SIRT1 is an NAD^+^-dependent deacetylase that serves as a key regulator of hepatic metabolic homeostasis by coordinating lipid metabolism and mitochondrial homeostasis [55]. Previous studies have demonstrated that hepatocyte-specific deletion of SIRT1 impairs PPARα signaling and fatty acid β-oxidation, resulting in hepatic steatosis and inflammation under HF diet conditions, whereas SIRT1 activation promotes PGC1α-dependent mitochondrial biogenesis and metabolic adaptation [55,56]. In addition, emerging evidence further suggests functional crosstalk between SIRT1 and Nrf2 signaling pathways in the regulation of cellular antioxidant responses and redox homeostasis [17,18]. In the present study, hepatic SIRT1 activity and mRNA expression were significantly reduced in HF-fed mice compared with NOR-fed controls, and this reduction was further exacerbated by vitamin D insufficiency in the HF + NVD group. Consistent with these changes, vitamin D insufficiency also decreased the hepatic NAD^+^/NADH ratio, suggesting an alteration in hepatic redox and metabolic status under conditions of dietary obesity. The concurrent reduction in the NAD^+^/NADH ratio and SIRT1 activity is biologically plausible given the well-established NAD^+^ dependence of SIRT1. Consistent with the present findings, Chang and Kim [41] also reported that vitamin D insufficiency was associated with reduced AMPK/SIRT1 activity in obese rats. Collectively, these observations support a potential association between inadequate vitamin D status and reduced hepatic SIRT1 activity during obesity. However, the present study does not establish that the altered NAD^+^/NADH ratio directly caused the reduction in SIRT1 activity, and additional mechanistic studies will be required to clarify this relationship. Moreover, hepatic mRNA expression of Nrf2 and several of its downstream antioxidant targets, including HMOX1, TXNRD, GSR, GPX, and SOD, was significantly reduced in the HF + NVD group compared with the HF group, suggesting a weakened transcriptional antioxidant response under vitamin D-insufficient conditions. Consistent with these observations, vitamin D has been shown to enhance Nrf2-mediated antioxidant defense in both in vivo and in vitro models. In obese rats with HF-induced NAFLD, the active form of vitamin D promoted Nrf2-mediated antioxidant genes, including GCLC, NQO1, SOD2, and CAT, whereas in hepatocyte models vitamin D attenuated oxidative stress through activation of the Nrf2 signaling pathway [57,58]. The present findings extend these observations by showing that dietary vitamin D insufficiency is accompanied by reduced SIRT1 activity and lower expression of Nrf2-related antioxidant genes during chronic HF feeding. Future studies employing genetic or pharmacological modulation of SIRT1 and/or Nrf2 will be necessary to determine whether these molecular alterations play a causal role in mediating the hepatic effects of vitamin D insufficiency. Importantly, these concurrent changes represent correlative findings and do not demonstrate direct regulation of Nrf2 by SIRT1 or establish the SIRT1–Nrf2 pathway as the causal mediator of hepatic steatosis. Rather, they support a working hypothesis that reduced SIRT1 activity and an insufficient Nrf2-related antioxidant response may contribute to the greater hepatic vulnerability associated with dietary vitamin D insufficiency. From a translational perspective, these findings raise the possibility that suboptimal vitamin D status may be associated with greater hepatic vulnerability in the context of obesity and chronic overnutrition. However, whether correction of vitamin D insufficiency can prevent or attenuate NAFLD progression independently of adiposity and other metabolic risk factors requires confirmation in prospective human studies.

The present study has several limitations. Only male C57BL/6J mice were included; therefore, potential sex-dependent responses were not evaluated. In addition, although the HF diet-induced C57BL/6J mouse model reproduces several key features of human obesity-associated NAFLD, findings from this model cannot be directly extrapolated to humans. Furthermore, although alterations in hepatic SIRT1 activity, Nrf2-related antioxidant gene expression, and related molecular markers were observed, direct mechanistic interactions between SIRT1 and Nrf2 were not experimentally examined using pathway-specific gain- or loss-of-function, genetic, pharmacological, or rescue approaches. Therefore, the concurrent SIRT1- and Nrf2-related alterations should be interpreted as correlative evidence supporting a working hypothesis rather than as confirmation of an established causal pathway. Moreover, mitochondrial status was evaluated primarily using molecular and morphological markers, including mtDNA content, TEM observation, and the expression of CPT1α and PPARα, rather than direct functional assays. Therefore, these findings should be interpreted as evidence of mitochondrial-related alterations rather than definitive evidence of impaired mitochondrial function. Future studies incorporating functional assessments, such as mitochondrial respiration, oxygen consumption rate, ATP production, or enzymatic activity, will be necessary to determine whether dietary vitamin D insufficiency directly impairs mitochondrial function. Finally, the absence of a NOR + NVD group limited our ability to distinguish the independent effects of dietary vitamin D depletion from those of obesity on vitamin D status. Inclusion of this group in future studies would strengthen the interpretation of these findings. Taken together, our findings demonstrate that dietary vitamin D insufficiency is associated with aggravated hepatic steatosis, oxidative injury, and alterations in molecular and morphological markers related to mitochondrial homeostasis in HF-fed mice. These hepatic abnormalities were accompanied by reduced SIRT1 activity and lower expression of Nrf2-related antioxidant genes. Although these concurrent alterations support a working hypothesis involving SIRT1- and Nrf2-related responses, further gain- and loss-of-function studies are required to determine whether these pathways causally contribute to the hepatic effects associated with dietary vitamin D insufficiency.

## 5. Conclusions

The present study demonstrates that vitamin D insufficiency exacerbates HF-induced hepatic steatosis and oxidative stress in obese mice. Vitamin D insufficiency was associated with increased hepatic mRNA expression of lipogenic genes and reduced expression of fatty acid β-oxidation-related genes, which may contribute, at least in part, to the observed hepatic lipid accumulation. In addition, vitamin D insufficiency further decreased hepatic SIRT1 activity and mRNA levels and the NAD^+^/NADH ratio while suppressing Nrf2 and its downstream antioxidant genes, suggesting impaired hepatic antioxidant defense and redox homeostasis under conditions of dietary obesity. Collectively, these findings indicate that dietary vitamin D insufficiency is associated with aggravated obesity-associated hepatic dysfunction accompanied by alterations in the hepatic SIRT1–Nrf2 pathway. Although the present findings support a potential role of this pathway in the hepatic response to vitamin D insufficiency, they do not establish a direct causal relationship, and further mechanistic studies are warranted. Overall, maintaining adequate vitamin D status may represent a promising nutritional strategy for reducing susceptibility to obesity-associated NAFLD.

## Data Availability

The original contributions presented in the study are included in the article/Supplementary Materials; further inquiries can be directed to the corresponding author.

## Supplementary Materials

The supporting information can be downloaded at https://www.mdpi.com/article/10.3390/nu18152481/s1, Table S1. Primers used for real-time quantitative polymerase chain reaction (RT-qPCR).
