# Supplementary material for: Dietary Vitamin D Insufficiency Aggravates High-Fat Diet-Induced Hepatic Steatosis Associated with Reduced Hepatic SIRT1 Activity and Nrf2-Related Antioxidant Gene Expression in Male C57BL/6J Mice
Source: Nutrients. 2026 Jul 31;18(15):2481. doi: 10.3390/nu18152481 (PMC13468119; doi:10.3390/nu18152481)
Supplement: Supplementary file 1 [file nutrients-18-02481-s001.zip › nutrients-4427942-supplementary.pdf]

Table S1. Primers used for real-time quantitative polymerase chain reaction (RT-qPCR).

Primer symbols	NCBI reference sequence accession number	Forward sequence (5′-3′)	Reverse sequence (5′-3′)	Product size (bp)
β-actin	NM_007393.5	GGA CCT GAC AGA CTA CCT CA	GTT GCC AAT AGT GAT GAC CT	208
CPT1α	NM_013495.2	GTG TTG GAG GTG ACA GAC TT	CAC TTT CTC TTT CCA CAA GG	100
FAS	NM_007988.3	AAC TCA CTG GCA GAA GAG AA	CTT CAA GAA GAT AGC CAT GC	134
GPX	NM_001329528.1	GAG GGT AGA GGC CGG ATA AG	TCT GCA GAT CGT TCA TCT CG	241
GSR	NM_010344.4	CAC GAC CAT GAT TCC AGA TG	CAG CAT AGA CGC CTT TGA CA	161
HMOX1	NM_010442.2	CAC GCA TAT ACC CGC TAC CT	CCA GAG TGT TCA TTC GAG CA	175
LDH	NM_010699.2	CCG TTA CCT GAT GGG AGA GA	GTA GGC ACT GTC CAC CAC CT	208
NRF1	NM_001164226.1	AAG TAT TCC ACA GGT CGG GG	TGG TGG CCT GAG TTT GTG TT	238
Nrf2	NM_010902.4	CTC GCT GGA AAA AGA AGT GG	CCG TCC AGG AGT TCA GAG AG	240
NQO1	NM_008706.5	TTC TCT GGC CG ATT CAG AGT	GGC TGC TTG GAG CAA AAT AG	180
PPARα	NM_011144.6	GAG GGT TGA GCT CAG TCA GG	GGT CAC CTA CGA GTG GCA TT	161
SIRT1	NM_019812.3	TAC CCC ATG AAG TGC CTC AA	AAC CAA TTC CTT TTG TGG GC	203
SOD	NM_011434.2	CCA GTG CAG GAC CTC ATT TT	CAC CTT TGC CCA AGT CAT CT	216
SREBP1c	NM_001358315.1	GCC TGC TTG GCT CTT CTC TT	AGG TCA GCT TGT TTG CGA TG	102
Tfam	NM_009360.4	GAG GCC AGT GTG AAC CAG TG	GCT CTG AAG CAC ATG GTC AA	158
TXNRD	NM_015762.2	CAG CGA GGA GAC CAT AGA GG	GCA CAT TGG TCT GCT CTT CA	155

CPT1α, carnitine palmitoyltransferase 1α; FAS, fatty acid synthase; GPX, glutathione peroxidase; GSR, glutathione reductase; HMOX1, heme oxygenase 1; LDH, lactate dehydrogenase; NRF1, nuclear respiratory factor 1; Nrf2, nuclear factor, erythroid derived 2, like 2 (Nfe2l2; NQO1, NAD(P)H dehydrogenase, quinone 1; PGC1α, peroxisome proliferative activated receptor gamma coactivator 1α; PPARα, peroxisome proliferator-activated receptor α; SIRT1, sirtuin 1; SOD, superoxide dismutase; SREBP1c, sterol regulatory element-binding protein-1c; Tfam, mitochondrial transcription factor A, TXNRD, thioredoxin reductase
